# Mental health among clients of the Sydney Medically Supervised Injecting Centre (MSIC)

**DOI:** 10.1186/s12954-016-0117-y

**Published:** 2016-10-12

**Authors:** Mark Goodhew, Allison M. Salmon, Christina Marel, Katherine L. Mills, Marianne Jauncey

**Affiliations:** 1 Sydney Medically Supervised Injecting Centre, PO Box 293, Kings Cross, Sydney, NSW 1340 Australia; 2 Centre of Research Excellence in Mental Health and Substance Use, National Drug and Alcohol Research Centre (NDARC), University of New South Wales, Sydney, Australia

**Keywords:** Mental health, Mental disorders, Supervised injecting centres, supervised injecting facilities, illicit drugs

## Abstract

The Sydney Medically Supervised Injecting Centre (MSIC) is a supervised injecting facility (SIF) where people who inject drugs (PWID) can do so legally, under health professional supervision. The majority of clients have low levels of education and employment, high rates of incarceration and unstable housing and poor social networks, and 70 % do not access local health services. These factors increase the risk of poor mental health, and it has been documented that PWID have elevated rates of mood, anxiety, personality and psychotic disorders; post-traumatic stress disorder (PTSD); and higher rates of trauma exposure, suicidality and self-harm. The current study is the first to investigate the mental health among clients of a SIF. Validated instruments to examine clients’ mental health, social networks and trauma histories were administered to 50 frequently attending clients by a mental health nurse. The majority of respondents were unemployed, homeless and had a history of incarceration, and 82 % report they had been diagnosed with a mental health problem, but only 24 % report they were receiving treatment. Respondents had poor social networks, had poorer mental health symptoms compared to US inpatients and had experienced multiple traumatic events, and a high number of respondents had scores indicative of PTSD. These results highlight the need for mental health clinicians to be employed in SIFs and other drug consumption rooms (DCRs) to assist clients to address their mental health and psychosocial needs, particularly in light of the fact that these services are often the only places these PWID engage with in an ongoing way.

## Background

The Sydney Medically Supervised Injecting Centre (MSIC) is a supervised injecting facility (SIF) where people who inject drugs (PWID) do so legally, under health professional supervision. Operational since May 2001, MSIC aims to reduce death and injury from drug overdose and reduce harm associated with injecting drug use. As in May 2015, MSIC had supervised more than 930,000 injections and managed over 5925 overdoses without a fatality. MSIC has more than 15,000 registered clients, of which approximately 600 clients attend in a typical month and 70 % of whom had not accessed local health services prior to MSIC registration [1, 2]. Engaging this “hard-to-reach” population, staff aim to enhance access to drug treatment and psychosocial and health services, including mental health, with over 11,500 referrals provided as in May 2015.

The broad population of PWID is characterised by low educational attainment and employment rates [3] and high rates of incarceration and unstable housing [4]. Such attributes are exaggerated among MSIC’s clients [2], of whom 92 % report unemployment and 65 % report unstable housing [4]. Additionally, PWID commonly have limited social networks, as rejection by non-using friends [5] often leads to social isolation [6], a well-documented risk factor for poor mental health [7]. Such social determinants of health are associated with mental health problems [8, 9], and consistent with these associations, PWID have documented elevated rates of mood, anxiety, personality and psychotic disorders [10, 11]; post-traumatic stress disorder (PTSD) [12]; and suicidality and self-harm [10, 12]. Trauma exposures such as being witness to serious injury or death, being involved in a life-threatening accident, being threatened with a weapon, being held captive or kidnapped [13] and being sexually abused as a child [14] are commonly experienced by people with substance dependence. These traumas usually occur before the onset of substance abuse disorders [13] and increase the risk of later mental health problems [14].

Despite high rates of mental health problems, PWID often encounter multiple barriers to accessing relevant services, ranging from clinician attitudes to the systems within which they work [15, 16]. Early evaluation of MSIC found that of those PWID registered to use the service, only 42 % were clients of local services targeted to their needs [2]. Likewise, qualitative research suggests substantial barriers to accessing treatment among MSIC clients, including unwillingness precipitated by stigma and discrimination [17]. MSIC clients are a hard-to-reach, and sometimes invisible, population who are unlikely to be captured by previous investigations of the mental health of people who use drugs.

By attracting a disengaged population, and offering services which facilitate sustained client contact, MSIC is uniquely placed to assess and engage with PWID regarding mental health. The present study builds on our formative research [17] by utilising validated instruments to examine clients’ mental health, social networks and trauma histories. There are now approximately 100 SIFs and drug consumption rooms (DCRs) around the world, and this study is the first to apply a structured, quantitative approach to mental health assessment among clients of these services.

## Methods

The study was approved by the Human Research Ethics Committee of South Eastern Sydney Local Health District. MSIC’s mental health nurse (author MG) assessed the mental health of 50 frequent attendees, defined as the 100 clients visiting most often between October and December 2014 (visit count range 29–321). It should be noted that within a typical month approximately 600 individual clients make up the majority of all visits to the service [1]. The first 50 of these 100 clients to present all agreed to participate and were reimbursed with an AU$40 voucher.

Structured questionnaires assessed demographic characteristics. Unstable accommodation was defined as primary (“sleeping rough”), secondary (staying with friends/relatives or in specialist homelessness services) and tertiary (neither secure lease nor private facilities) homelessness [4]. A broad mental health history was collected, including suicide, self-harm, previous mental health diagnoses, treatment and prescription of psychiatric medication. Lubben’s Social Network Scale-6 (LSNS-6) was used to assess perceived social support from family and friends [18]. The Behavior and Symptom Identification Scale (BASIS-24) provided a measure of recent difficulty in the symptom and functioning domains that underlie the need for mental health services [19]. BASIS-24 scores range from 0 to 5, with 5 the highest score indicating severe mental health symptoms and functional difficulties [19]. Trauma exposure and PTSD were assessed with the Composite International Diagnostic Interview (CIDI) version 2.1 and the PTSD Checklist (PCL-C), respectively [20, 21]. The CIDI measures lifetime and childhood exposure to traumatic events [20], and the PCL-C 17 items are added to obtain a possible score range from 17 to 85, and a cut-off of 50 is a predictor of a PTSD diagnosis [21].

## Results and discussion

Participants (*N* = 50) had a mean age of 42 years (SD 9.2); 70 % were male, 26 % female and 4 % transgender; and 92 % were unemployed. Sixteen percent of participants identified as Aboriginal and/or Torres Strait Islander; 62 % reported current unstable accommodation; and 70 % reported a history of incarceration.

Eighty-two percent reported that “a doctor had ever told (them) that (they) had a mental health problem” (Table 1). Self-reported diagnoses included the following: mood disorders including depression (48 %) and bipolar disorder (16 %); anxiety disorders including anxiety (36 %), panic disorder/attacks (4 %), obsessive-compulsive disorder (4 %) and generalised anxiety disorder (2 %); psychotic illnesses including schizophrenia (22 %), drug-induced psychosis (6 %) and schizoaffective disorder (4 %); PTSD (12 %); attention deficit hyperactivity disorder (10 %); and personality disorders including borderline personality disorder (4 %) and antisocial personality disorder (4 %). Among the 54 % of participants who reported a previous suicide attempt, a median of 2 attempts (range 1–12) had been made. One third of the sample reported a history of self-harm.

**Table 1:** Mental health indicators reported by MSIC frequently attending clients (*N* = 50)

Mental health indicator	% sample
Any mental health diagnosis by a doctor (lifetime)	82
Mood disorder (lifetime)	64
Anxiety disorder (lifetime)	46
Psychotic illness (lifetime)	32
Post-traumatic stress disorder (lifetime)	12
Attention deficit hyperactivity disorder (lifetime)	10
Personality disorder (lifetime)	8
History of suicide attempt/s	54
History of self-harm	44
Currently receiving support from mental health services	24

Just 24 % of participants reported currently receiving mental health treatment, including 8 % from a psychiatrist/psychiatric registrar and 2 % from a general practitioner. Forty-four percent reported current psychiatric medication prescription, including antipsychotics (20 %), antidepressants (20 %) and mood stabilisers (4 %). In this open-ended format (“Are you prescribed any psychiatric medications? Specify which”), no participants reported a current benzodiazepine prescription.

### Social networks, isolation and mental health symptoms

Mean score on the LSNS-6 was 9 (SD 6.2); 70 % received a score <12 indicative of social isolation. Mean total score on the BASIS-24 was 2.59 (SD 0.79), and Table 2 outlines the subscale means, which are compared to a US mental health inpatient population.

**Table 2:** BASIS-24 (mental health symptoms over the past week) (*N* = 50)

	MSIC		US inpatient	
	Mean	SD	Mean	SD
Total	2.59	0.79	1.85	0.83
Depression/functioning	2.64	1.00	2.22	1.13
Interpersonal problems	2.78	0.87	1.76	1.06
Self-harm	1.62	0.89	1.15	1.25
Emotional labiality	2.78	1.01	1.96	1.13
Psychosis	2.01	1.05	1.11	1.15
Substance abuse	3.03	0.62	1.85	0.83

### Trauma

Ninety-six percent of the sample had experienced a traumatic event in their lives (M 4.54, SD 2.45), including a mean of 3.04 traumatic exposures before the age of 16 (SD 2.50) (Table 2). Mean PCL-C score was 44.54 (SD 17.33), with 36 % scoring above 50, indicative of current PTSD.

This study, the first to apply a structured, quantitative approach to the assessment of mental health among clients of SIFs and DCRs, documented elevated lifetime rates of mental health disorders among Sydney MSIC clients. Over 80 % reported having ever received a mental health diagnosis from a doctor, most commonly mood (64 %), anxiety (46 %) and psychotic disorders (32 %). More than one half (54 %) reported attempted suicide and 34 % history of self-harm. These estimates are considerably higher than those in the Australian general population (46 % lifetime prevalence) [22] and at the upper end of lifetime prevalence estimates reported for Australians in substance use treatment (46–100 %) [23]. Further highlighting MSIC clients’ poor mental health were BASIS-24 scores, a measure of symptom and functioning difficulties in the preceding week. The mean BASIS-24 score for MSIC clients (2.59) was substantially higher than the benchmark figure for patients admitted to US mental health facilities (1.85), as were a number of the mean subscale scores including psychosis (2.01 vs 1.11) and substance use (3.03 vs 1.85) [19]. These scores highlight that MSIC clients have more severe mental health symptoms and functioning than patients within a mental health facility [19].

Results clearly suggest substantial mental health needs, yet 76 % of participants were not currently accessing mental health treatment. There was also substantial disconnect between MSIC clients reporting prescription of psychiatric medication (44 %) and having a qualified prescriber (10 %). Given the high rates of anxiety disorders among participants, the absence of prescription benzodiazepine use was unexpected. This result may reflect recent changes to benzodiazepine scheduling by Australia’s Therapeutic Goods Administration [24] and/or the normalisation of benzodiazepine use among MSIC clients and their ready illicit access to this class of drugs.

Given the robust relationships between mental health and social determinants including education, employment and housing [8, 9], and in light of the demographic characteristics of MSIC clients [2] and in this study, elevated prevalence of untreated mental health disorders among this group is unsurprising. Seventy percent of this sample was deemed socially isolated by the LSNS-6 [18]. As an integral component of health and well-being, MSIC clients’ lack of social connectedness is undoubtedly inextricably linked to their poor mental health [7]. Consistent with the literature, trauma exposure and PTSD as assessed by the PCL-C were highly prevalent [12]. Given that past trauma is strongly associated with mental health problems [14], the high rates of exposure of participants to traumatic events such as death, violence, sexual violence and natural disasters, including during childhood, are also consistent with the patterns of poor mental health among MSIC clients (Table 3). However, the discrepancy between the proportion of clients having ever received a diagnosis of PTSD (12 %) and the proportion screening positive for current PTSD on the PCL-C (50 %) indicates that there may be marked underdiagnosis of this disorder, and possibly other disorders, among clients of SIFs and DCRs.

**Table 3:** Lifetime and childhood exposure to traumatic events among MSIC frequently attending clients

Trauma	% ever experienced	% experienced prior to age 16 years
Witnessed someone badly injured or killed	78	42
Seriously physically attacked or assaulted	72	56
Threatened with a weapon, held captive or kidnapped	68	32
Involved in a life-threatening accident	58	30
Molested	52	46
Raped	42	40
Involved in a fire, flood or natural disaster	34	24
Tortured or victim of terrorist	34	26
Direct combat experience in a war	12	6

Events listed in the table are verbatim from the CIDI version 2.1 [20]

This cross-sectional study is unable to delineate the extent to which mental health problems among MSIC clients are a cause or a consequence of drug use. Another limitation of this study is its sample size of 50. In a previous study of street-based injectors in Kings Cross (MSIC’s location), indicators of social marginalisation, including unstable housing, unemployment and public injecting, were significantly associated with psychological distress, while indicators of drug use were not [25]. Regardless of the temporal sequencing of trauma, social isolation, mental health disorders and drug use, the fact remains that despite their reluctance to engage with other health services, clients suffer poor mental health. As a service that facilitates sustained, ongoing contact with clients, MSIC is uniquely placed to assess and engage with PWID around mental health issues. Indeed, this potential is reflected both in the visit numbers of the frequent attendees described here (up to 321 within a 3-month period) and in the 100 % response rate of clients invited to participate in this study.

## Conclusions

Specialised mental health services should be essential partners in the establishment of SIFs, and the ever-increasing number of DCRs, due to the high levels of mental distress among PWID and the multiple traumatic events they experience. Based on our findings, we recommend that, where possible, SIFs and DCRs implement multiple strategies to enhance mental health outcomes, including the following:

- Fostering good working relationships with local mental health services to create effective referral pathways
- Employment of a specialised mental health clinician
- Ongoing staff training in mental health, risk assessments and trauma informed care
- Establishing regular onsite psychiatric clinics for clients unwilling to access mainstream health services

## Abbreviations

BASIS-24: Behavior and Symptom Identification Scale
CIDI: Composite International Diagnostic Interview
DCRs: Drug consumption rooms
LSNS-6: Lubben’s Social Network Scale-6
MSIC: Sydney Medically Supervised Injecting Centre
PCL-C: PTSD Checklist
PTSD: Post-traumatic stress disorder
PWID: People who inject drugs
SIF: Supervised injecting facility
